# Supplementary material for: Ontogenetic Changes in the Chemical Profiles of Piper Species
Source: Plants (Basel). 2021 May 28;10(6):1085. doi: 10.3390/plants10061085 (PMC8227164; doi:10.3390/plants10061085)
Supplement: Supplementary file 1 [file plants-10-01085-s001.zip › plants-1207988-supplementary.pdf]

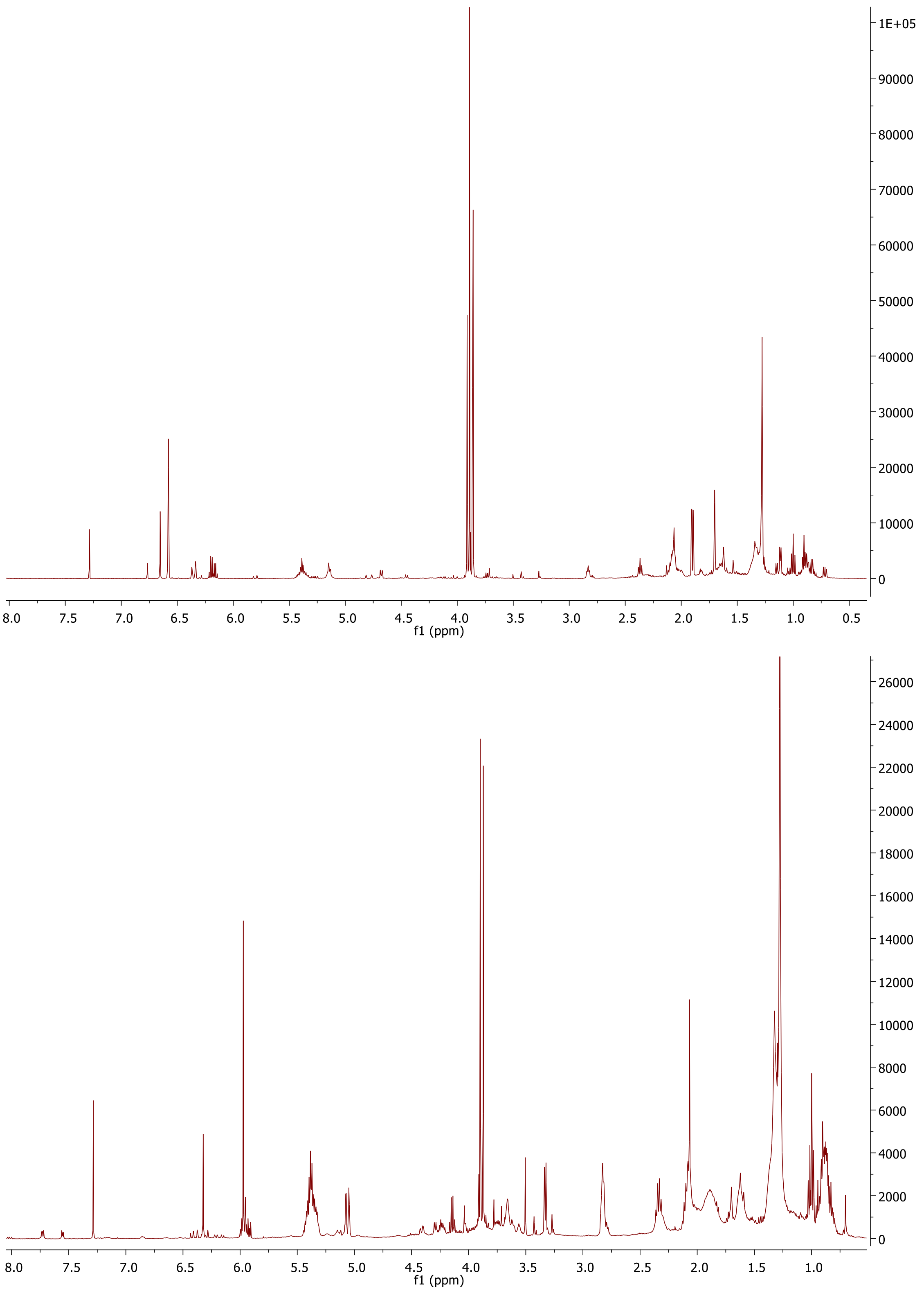

Supplementary Figure S1. ^{1}H NMR (500 MHz, $CDCl_3$) spectra of crude extracts from adult (top) and seedling (bottom) leaves of *P. solmsianum*.

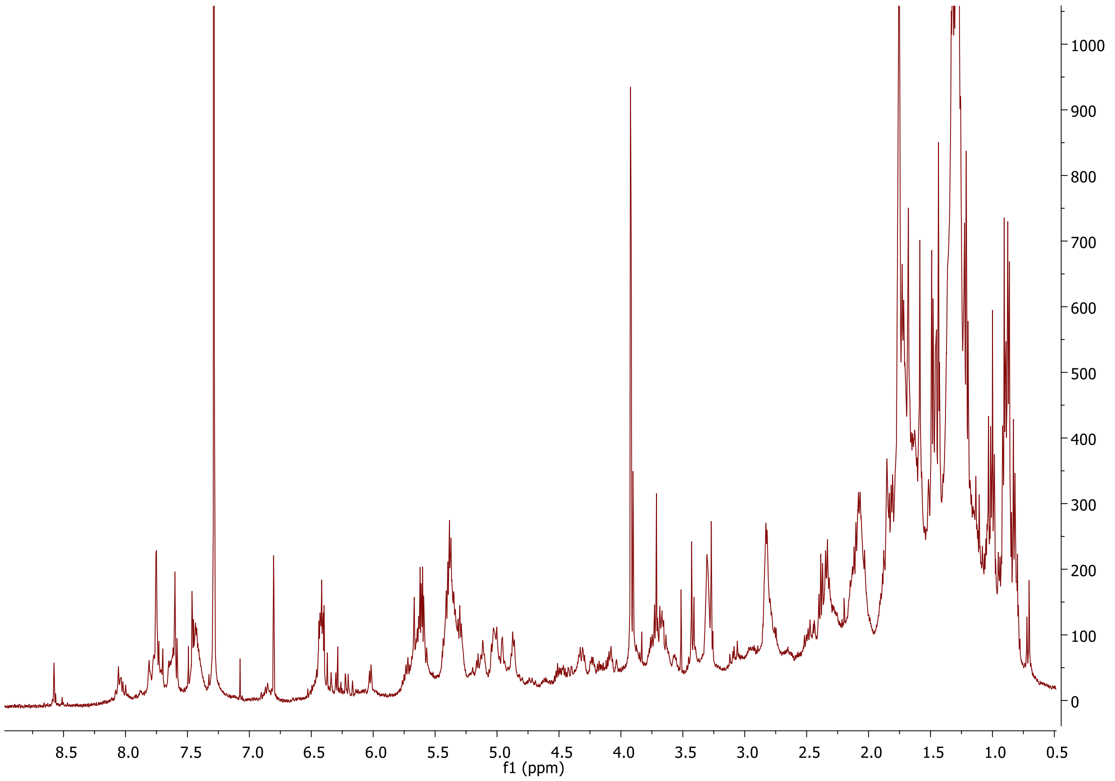

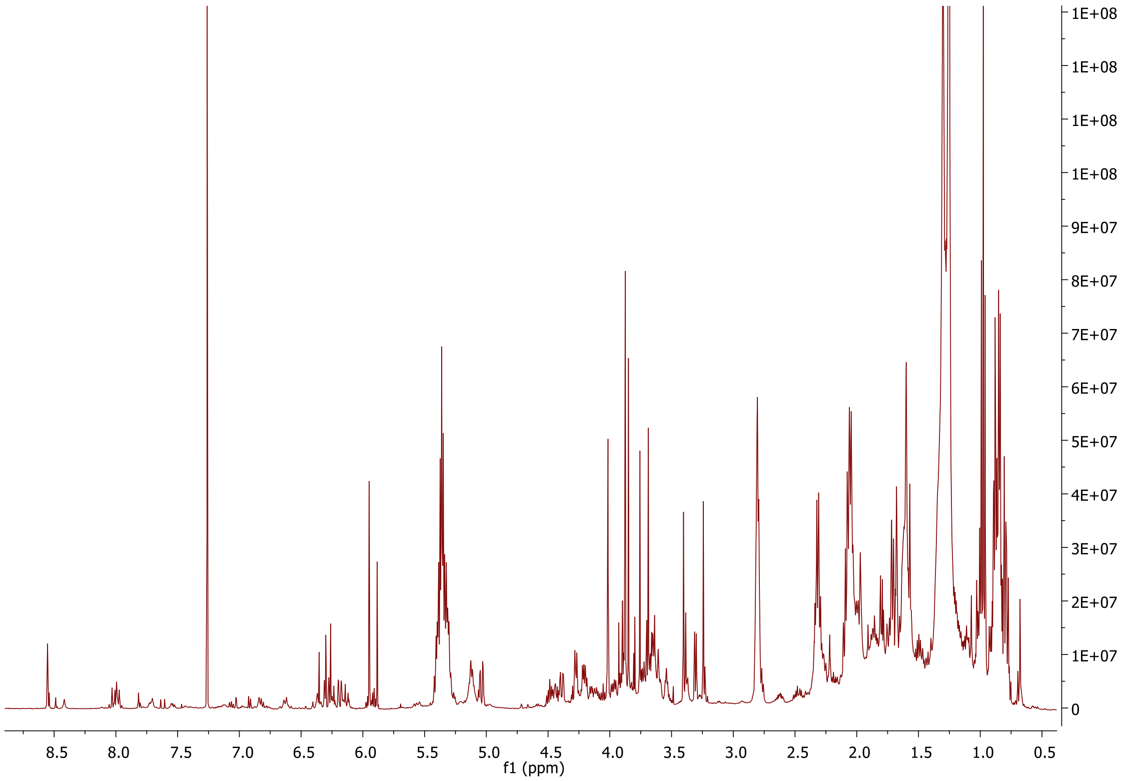

Supplementary Figure S2. ^{1}H NMR (500 MHz, $CDCl_3$) spectra of crude extracts from adult (top) and seedling (bottom) leaves of *P. gaudichaudianum*.

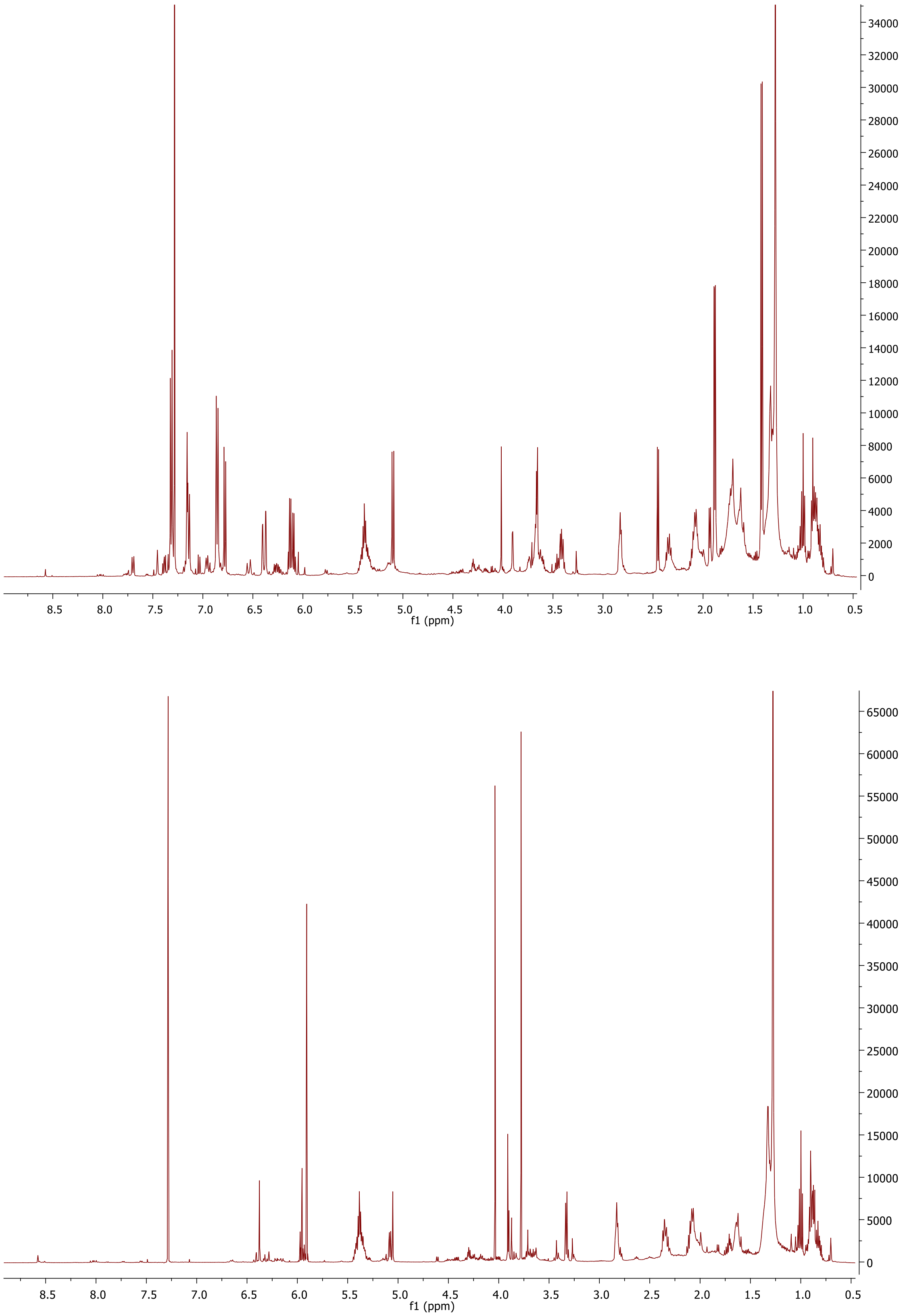

Supplementary Figure S3. ^{1}H NMR (500 MHz, $CDCl_3$) spectra of crude extracts from adult (top) and seedling (bottom) leaves of *P. regnellii*.

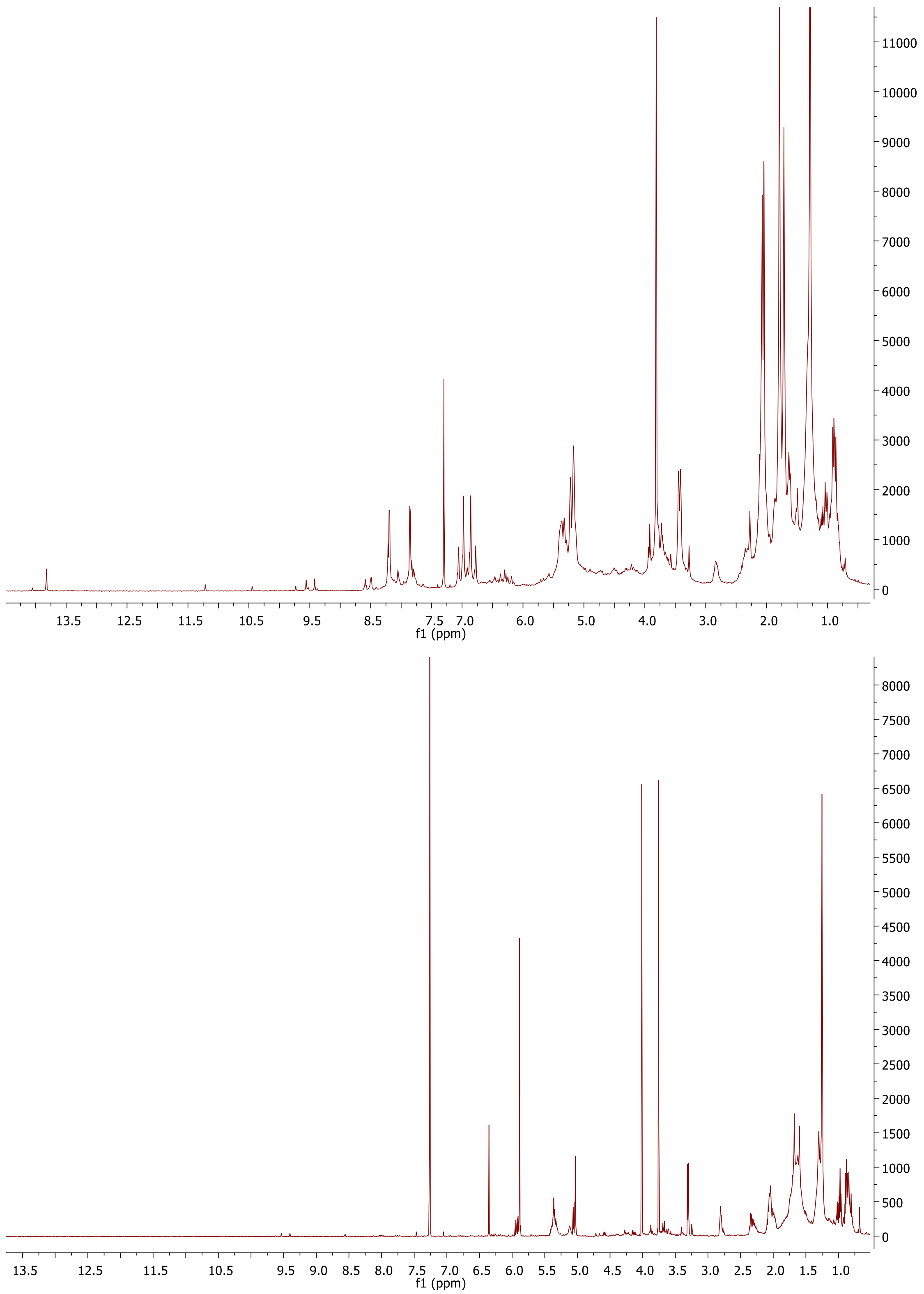

Supplementary Figure S4. ^{1}H NMR (500 MHz, $CDCl_3$) spectra of crude extracts from adult (top) and seedling (bottom) leaves of *P. hemmendorffii*.

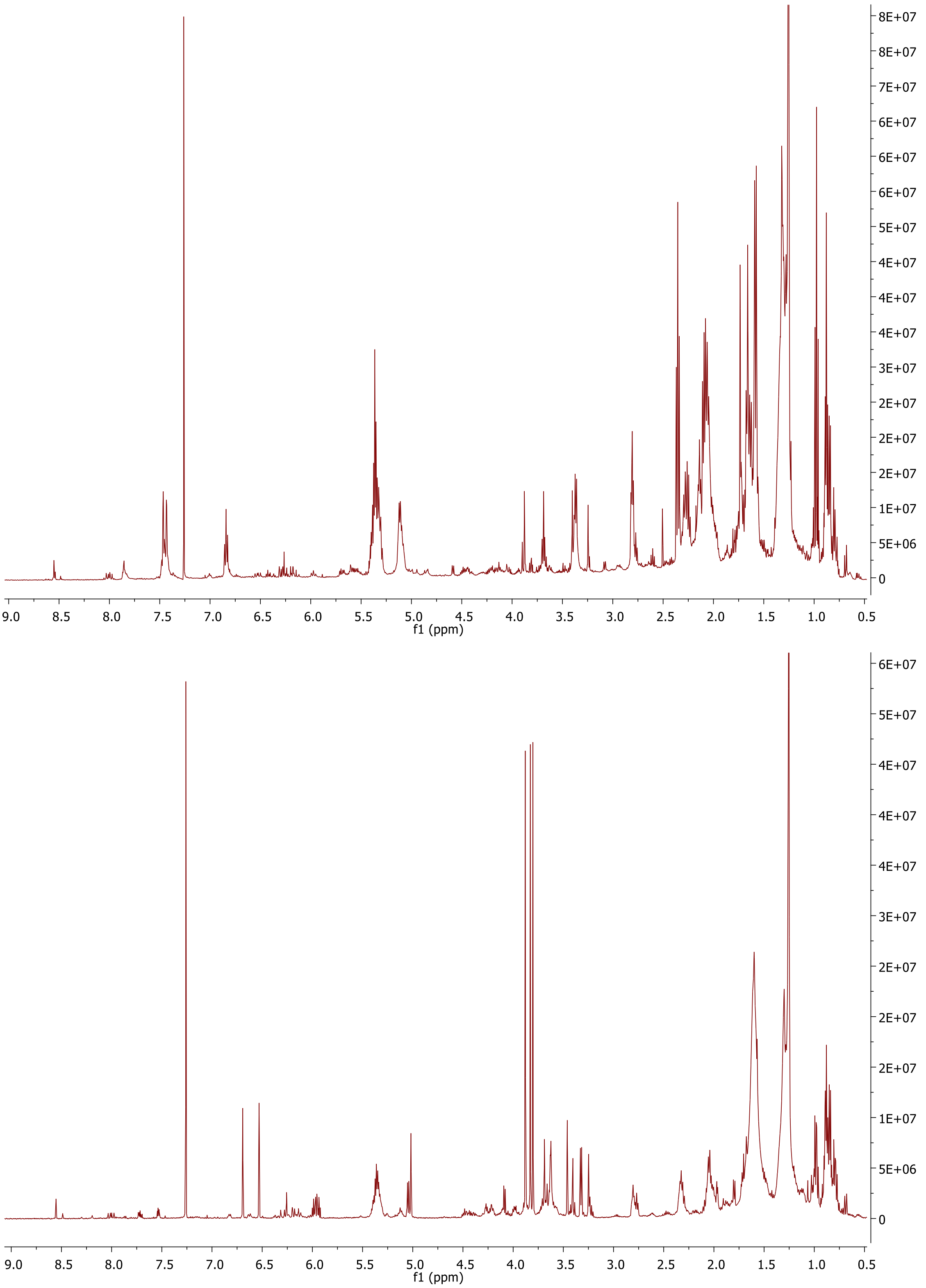

Supplementary Figure S5. ^{1}H NMR (500 MHz, $CDCl_3$) spectra of crude extracts from adult (top) and seedling (bottom) leaves of *P. caldense*.

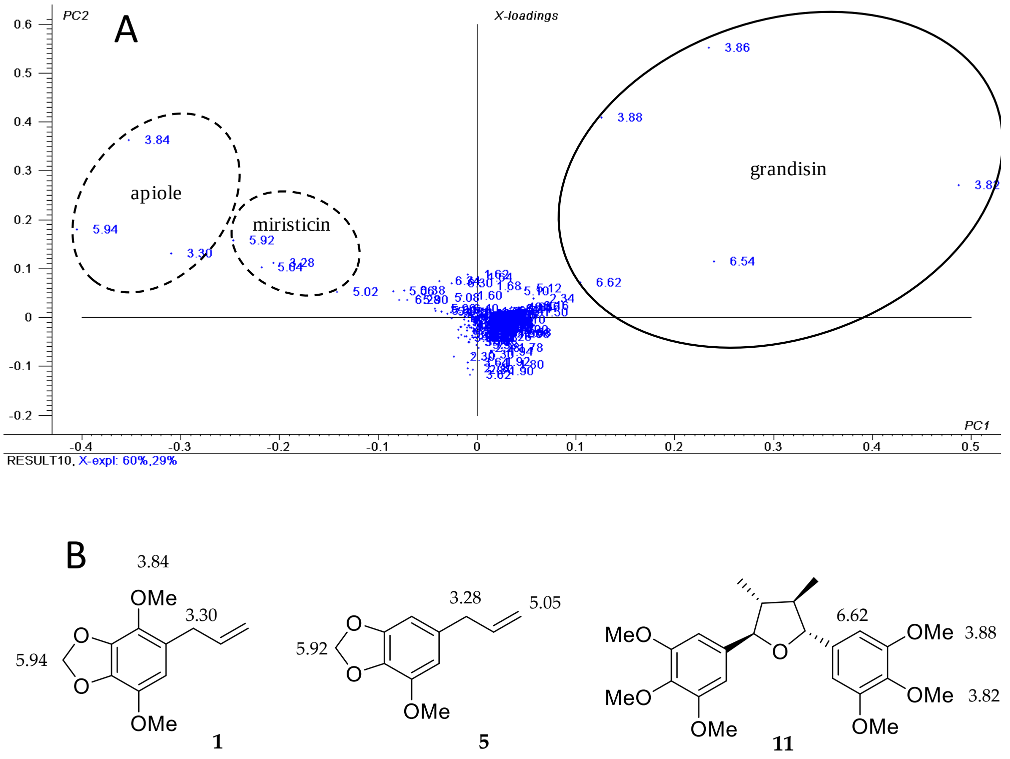

Supplementary Figure S6. Loading plot (A) obtained by PCA using ¹H NMR data of crude extracts of seedling leaves (at 3, 6, 9, 12 and 15 months) and adult leaves of *P. solmsianum*. Chemical structures with assignments of chemical shifts (B) of apiole (**1**), myristicin (**5**) and grandisin (**12**) observed in their ¹H NMR data.

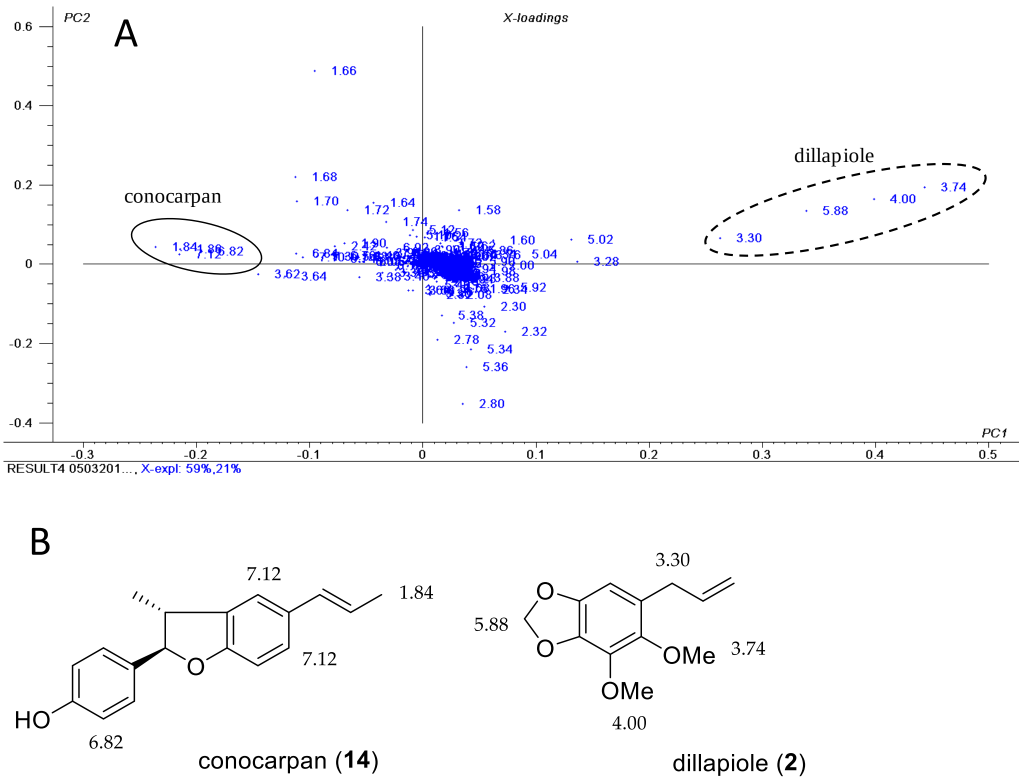

Supplementary Figure S7. Loading plot (A) obtained by PCA using NMR data of crude extracts of seedling leaves (seedlings at 3, 6, 9, 12 and 15 months) and adult leaves of *P. regnellii*. Chemical structures with assignments of chemical shifts (B) of conocarpan (**14**), and dillapiole (**2**) observed in their ^{1}H NMR data.

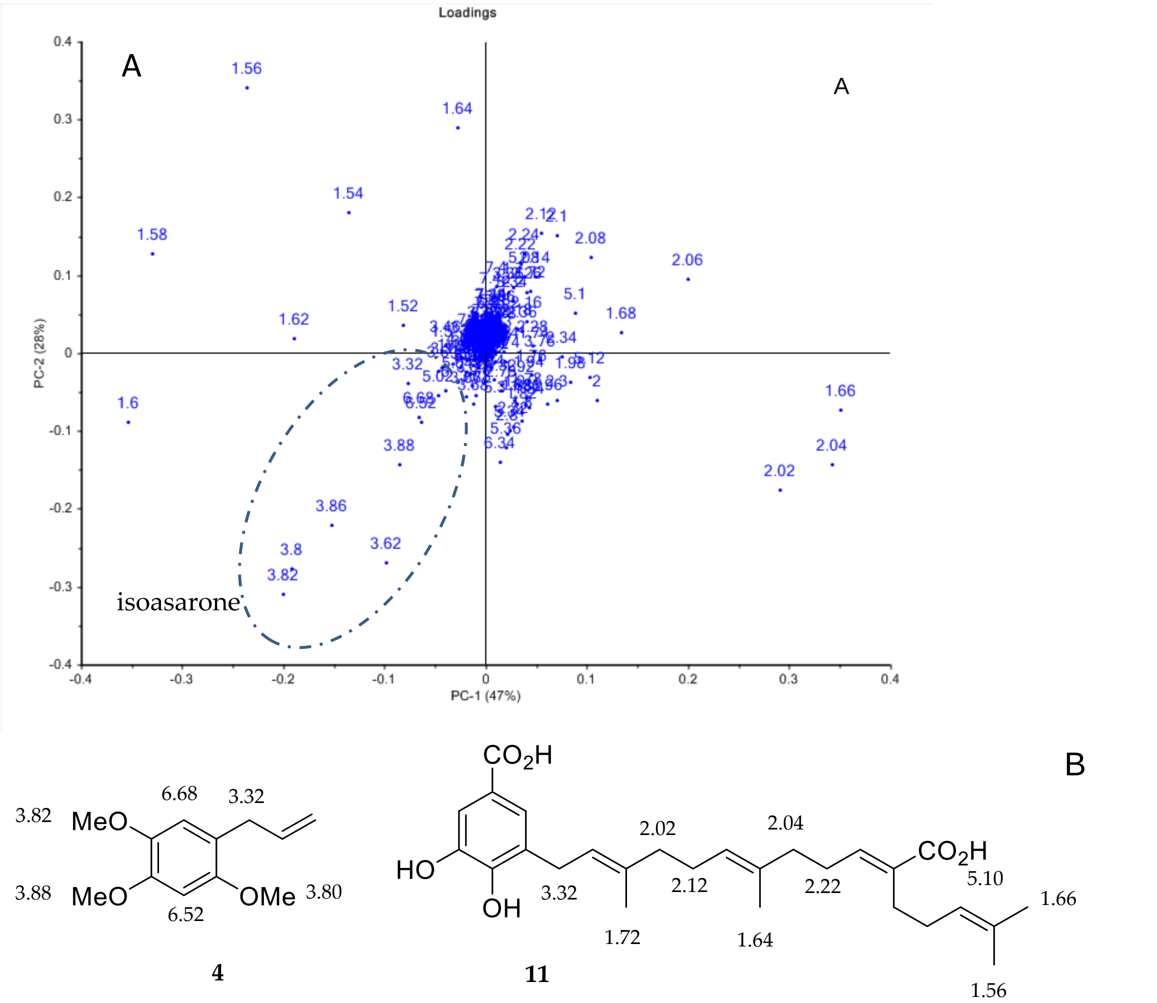

Supplementary Figure S8. Loading plot (A) obtained by PCA using NMR data of crude extracts of seedling leaves (seedlings at 3, 6, 9, 12 and 15 months) and adult leaves of *P. caldense*. Chemical structures with assignments of the main chemical shifts (B) of isoasarone (**4**), and caldensinic acid (**11**) observed in their ^{1}H NMR data.

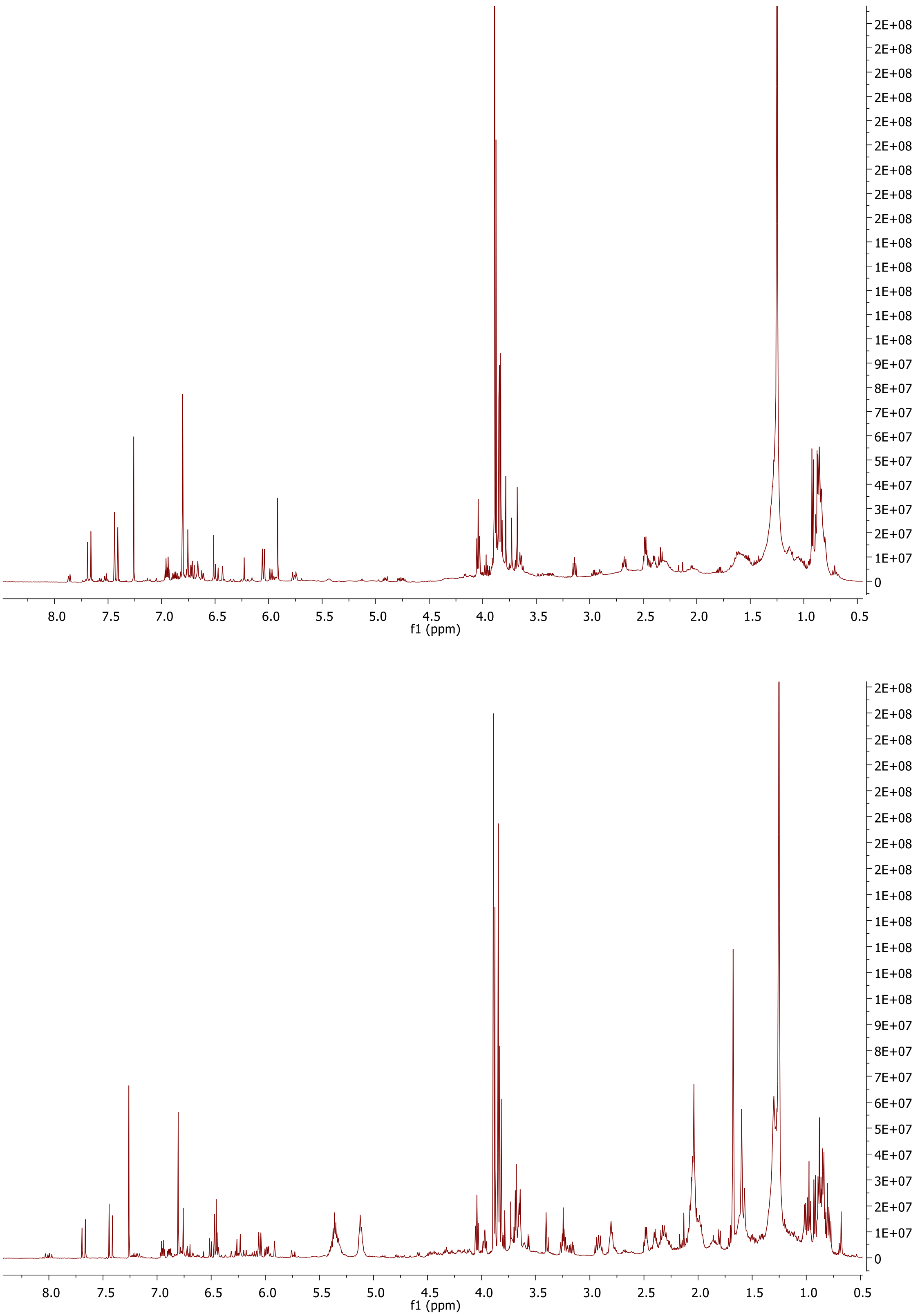

Supplementary Figure S9. ^{1}H NMR (500 MHz, $CDCl_3$) spectra of crude extracts from adult (top) and seedling (bottom) leaves *P. tuberculatum*.

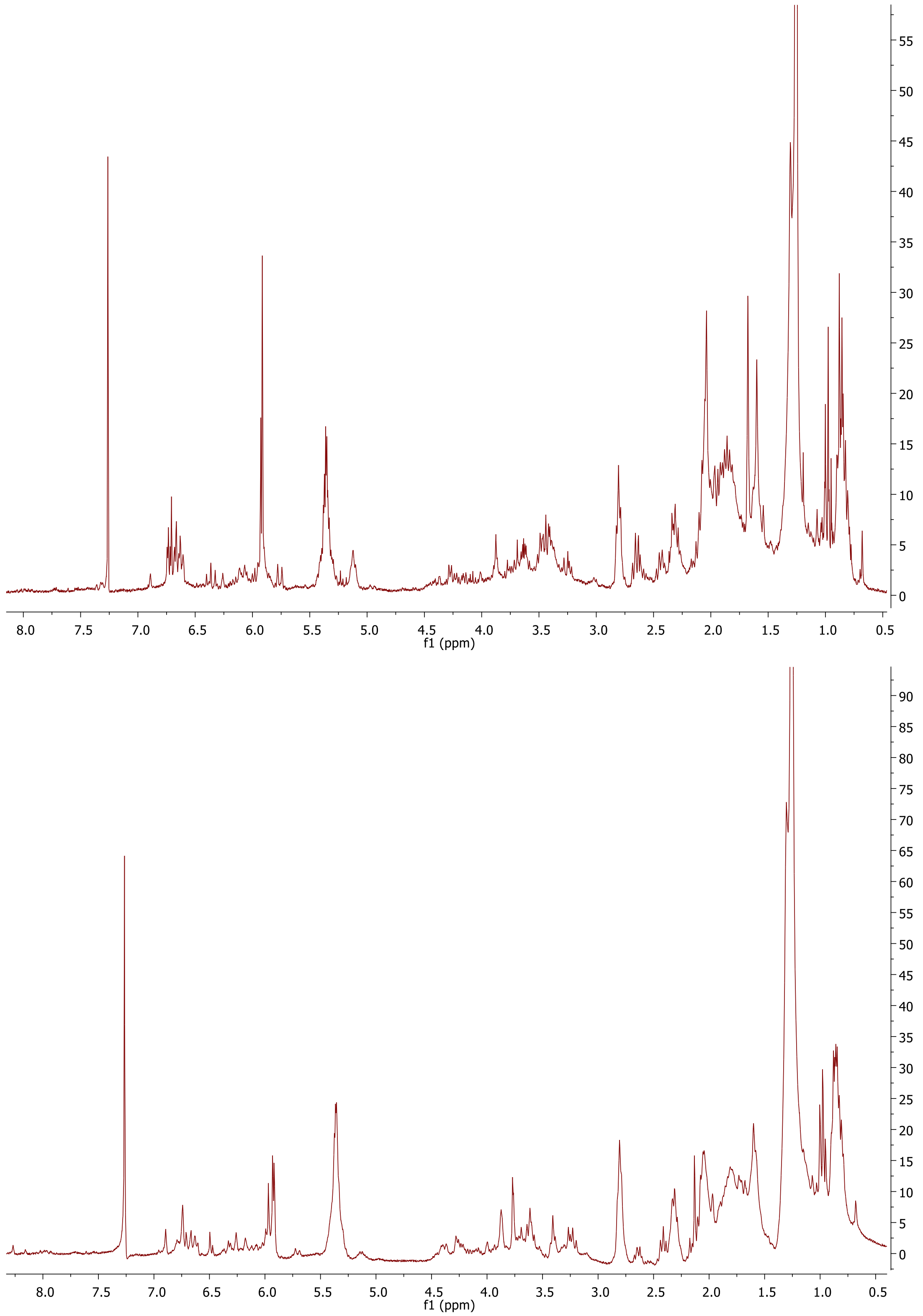

Supplementary Figure S10. ^{1}H NMR (500 MHz, $CDCl_3$) spectra of crude extracts from adult (top) and seedling (bottom) leaves of *P. amalago*.

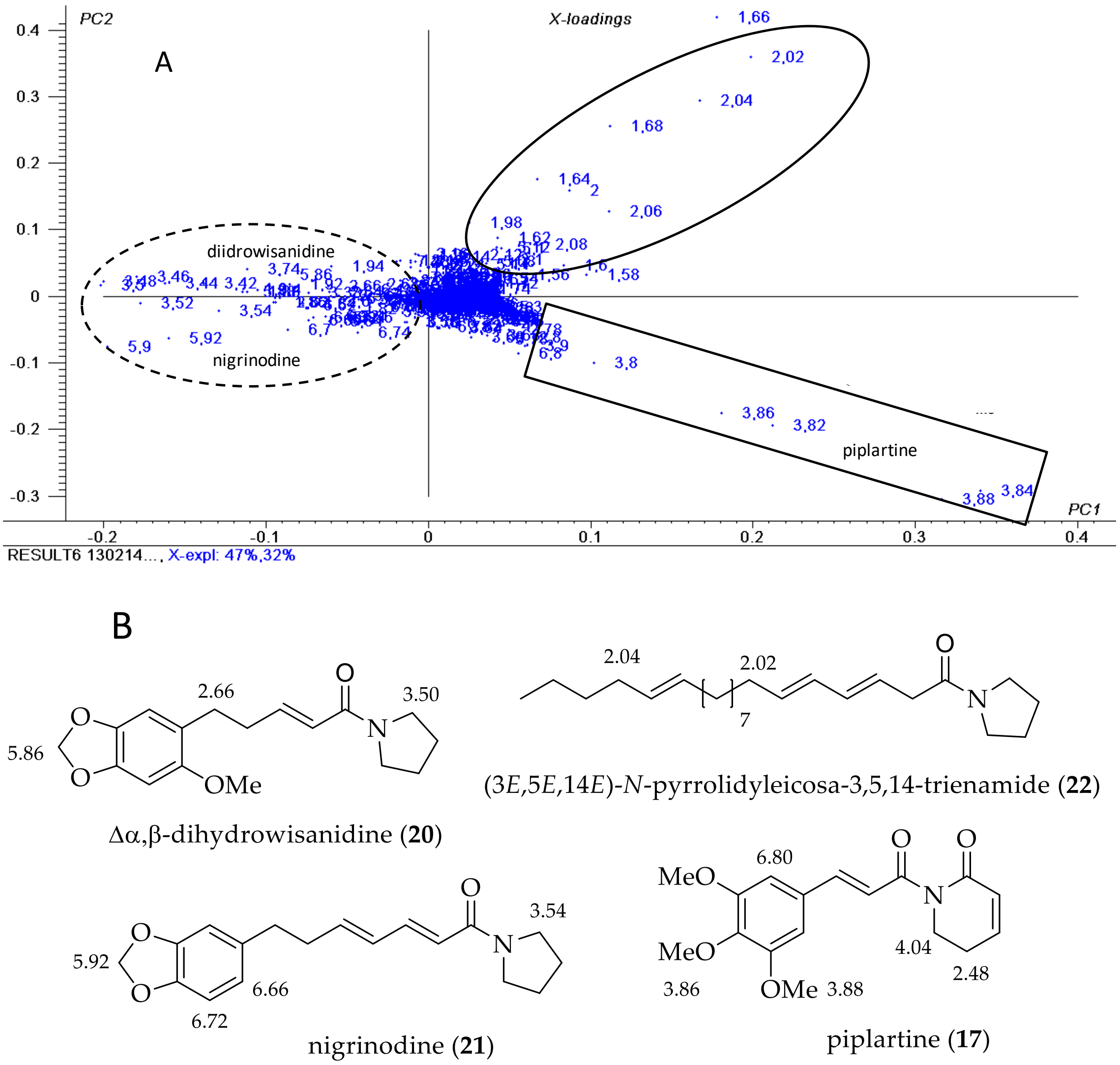

Supplementary Figure S11. loading plot (A) obtained by PCA of ^{1}H NMR data of crude extracts of seedling and adult leaves of *P. tuberculatum*, *P. amalago* and *P. reticulatum*. Chemical structures with assignment (B) of the main ^{1}H NMR chemical shifts of the amides piplartine (**17**), Δ*α*,β-dihydrowisanidine (**20**), nigrinodine (**21**) and (3*E*,5*E*,14*E*)-*N*-pyrrolidyleicosa-3,5,14-trienamide (**22**).

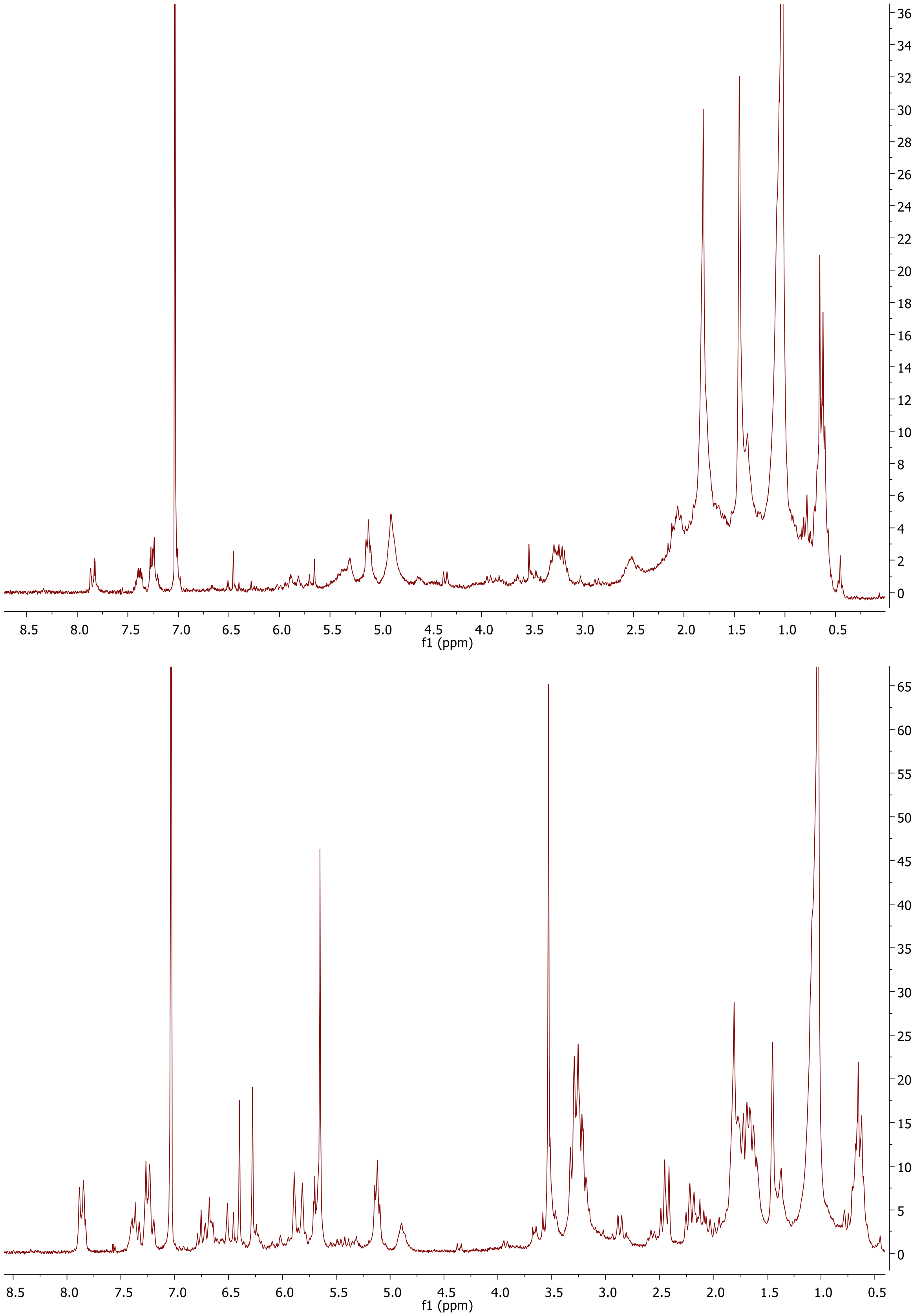

Supplementary Figure S12. ^{1}H NMR (500 MHz, $CDCl_3$) spectrum of crude extracts from adult (top) and seedling (bottom) leaves of *P. reticulatum*.

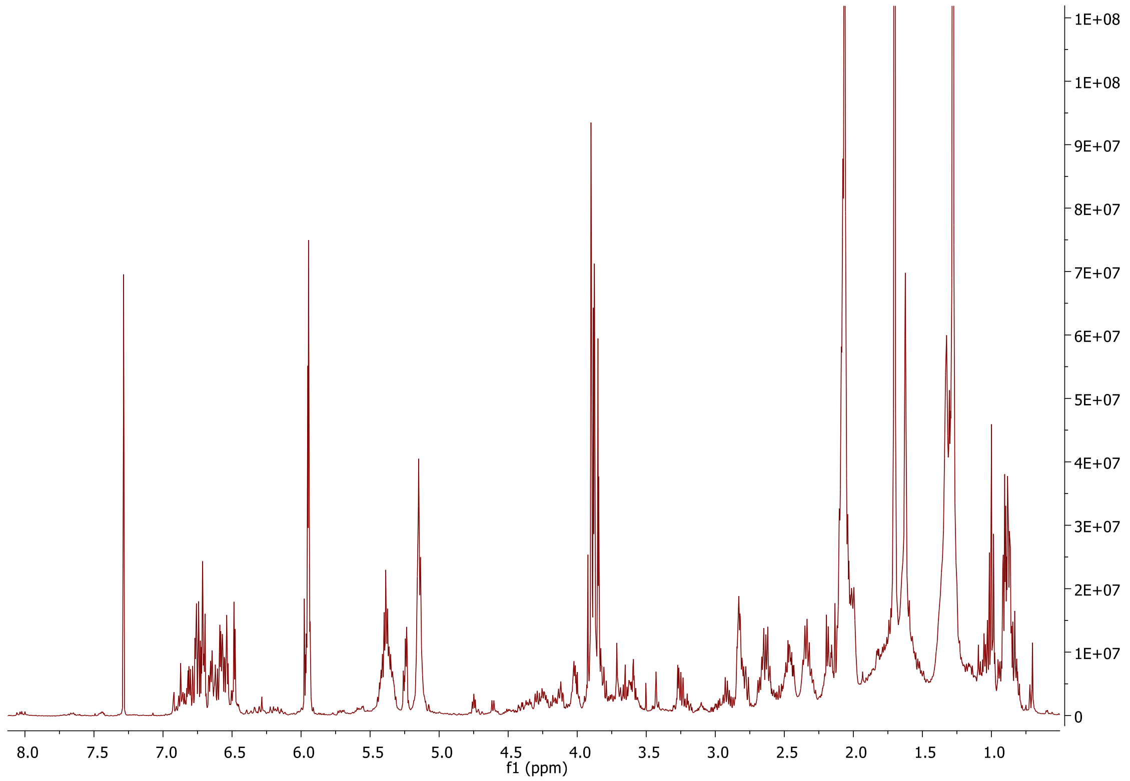

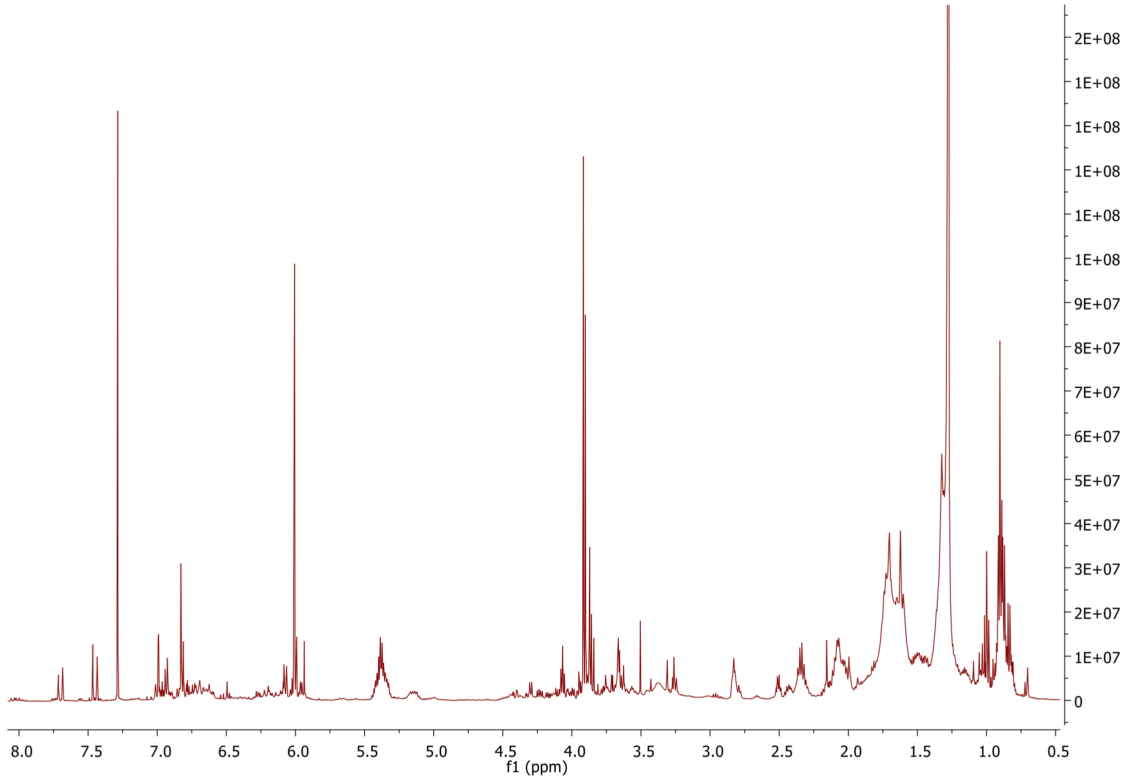

Supplementary Figure S13. ^1H NMR (500 MHz, $CDCl_3$) spectra of crude extracts from adult (top) and seedling (bottom) leaves of *P. richardiaefolium*.

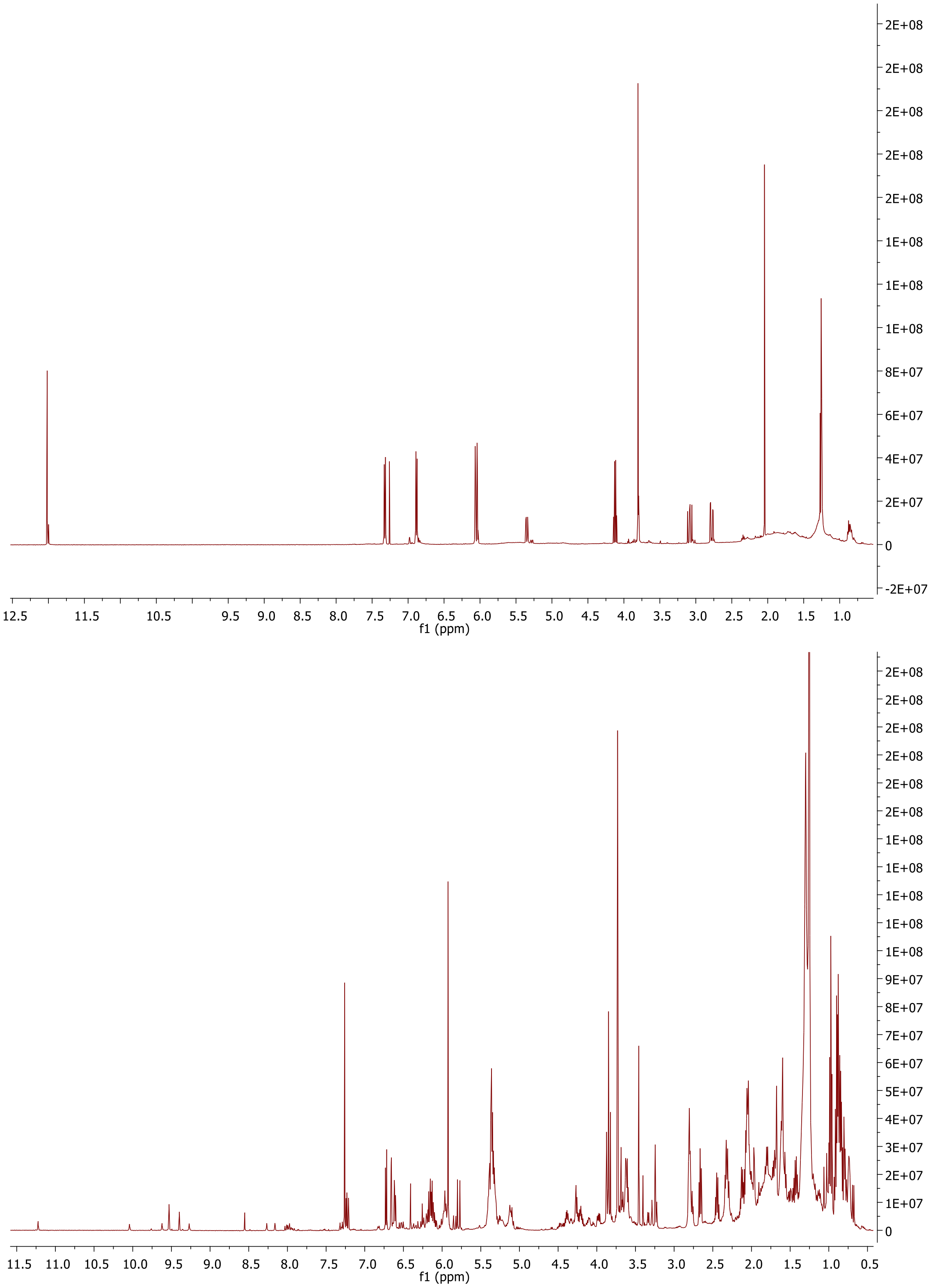

Supplementary Figure S14. ^{1}H NMR (500 MHz, $CDCl_3$) spectra of crude extracts from adult (top) and seedling (bottom) leaves of *P. permucronatum*.

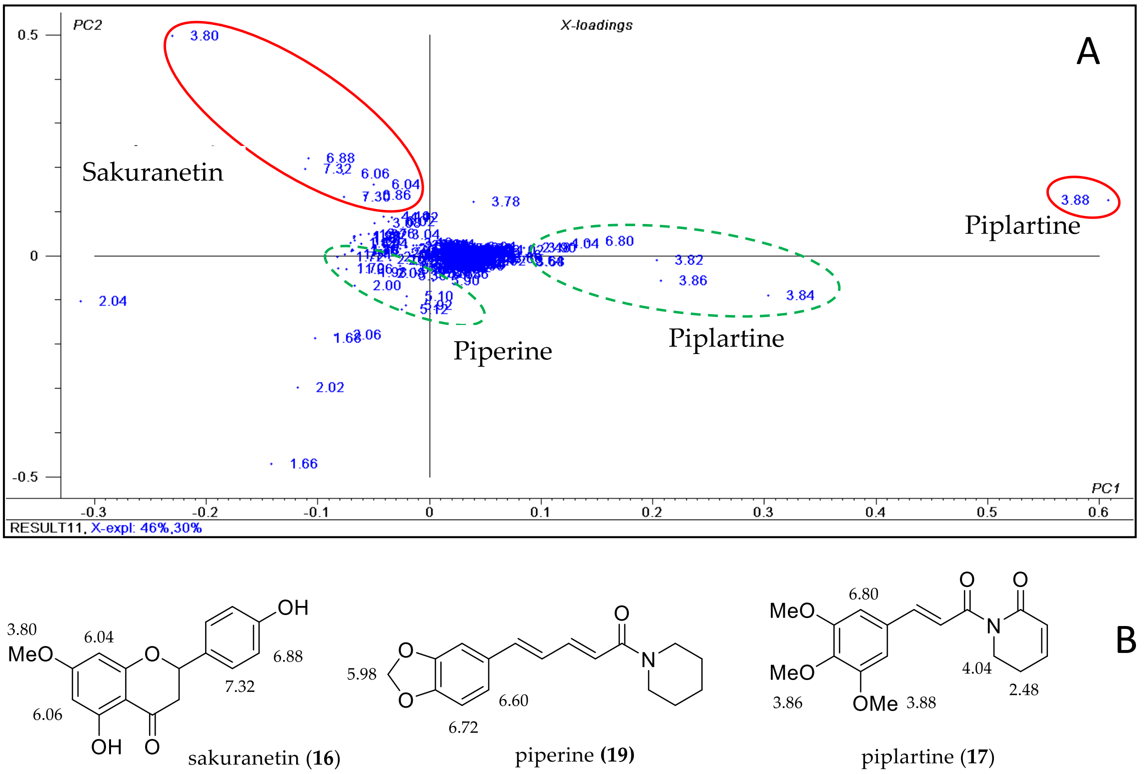

Supplementary Figure S15. Loadings (A) of the analysis of main components of the ^{1}H NMR data of the adult and seedling leaves of *P. permucronatum*, *P. richardiaefolium* and *P. tuberculatum*. Chemical structures and assignment (B) of the main ^{1}H NMR chemical shifts of sakuranetin (**16**), piperine (**19**) and piplartine (**17**).

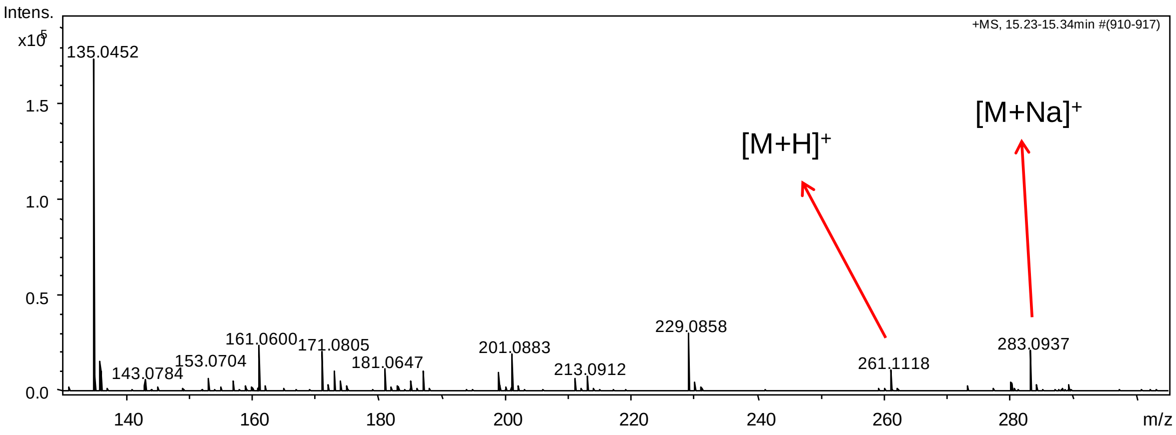

Supplementary Figure S16. HRESIMS spectrum of compound **23** isolated from seedling leaves of *P. permucronatum*.

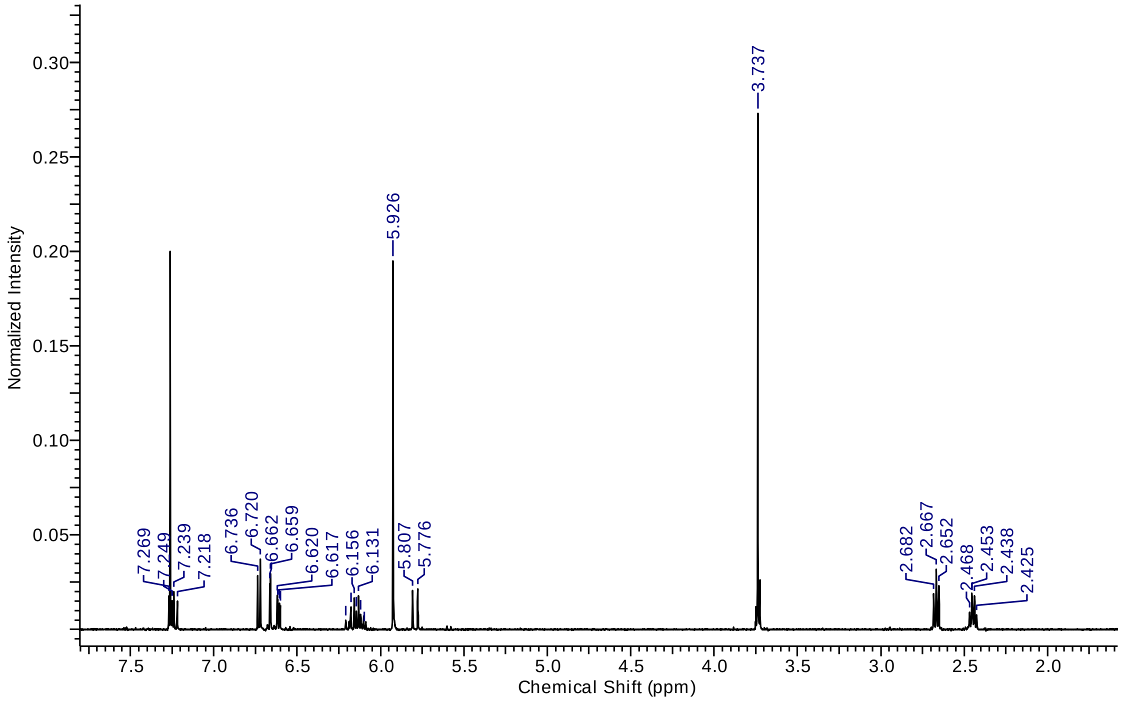

Supplementary Figure S17. ^{1}H NMR (500 MHz, $CDCl_3$) spectrum of compound **23** isolated from seedling leaves of *P. permucronatum*.

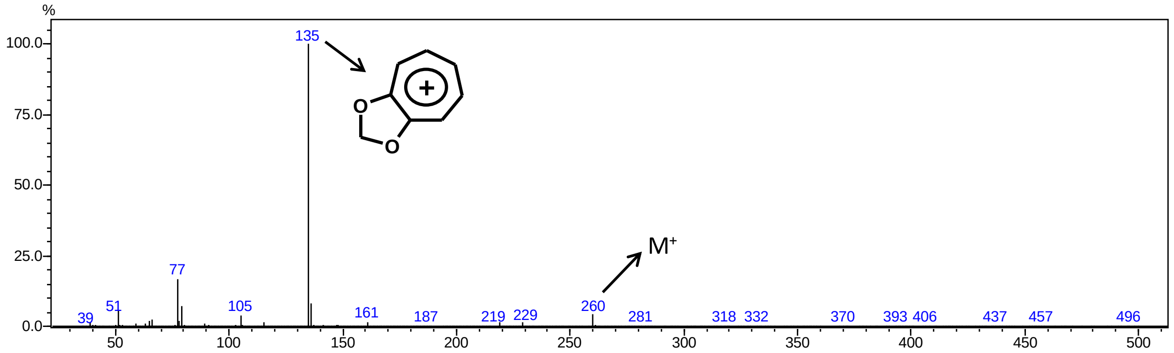

Supplementary Figure S18. EIMS spectrum of compound **23** isolated from seedling leaves of *P. permucronatum*.

Table S1. ^{1}H and ^{13}C NMR data for compound **23** isolated from seedlings leaves of *P. permucronatum*.

Position	δ ^{1}H, (*J* in Hz)	δ ^{13}C
1	-	134.9
2	6.66 (1H, d, 1.5)	108.8
3	-	147.4
4	-	146.1
5	6.73 (1H, d, 8.0)	108.2
6	6.61 (1H, dd, 8.0 and 1.5)	121.2
7	2.67 (2H, t, 7.5)	34.8
8	2.44 (2H, m)	35.0
9	6.14 (1H, m)	143.2
10	6.14 (1H, m)	129.0
11	7.25 (1H, dd, 15.0 and 10.0)	145.7
12	5.79 (1H, d, 15.0)	119.2
13	-	167.2
13-OMe	3.73 (3H, s)	51.5
O-CH$_2$-O	5.93 (2H, s)	100.8

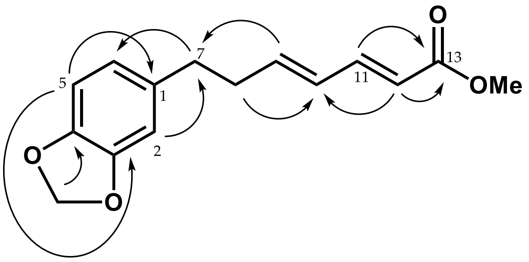

Figure S19. HMBC correlations observed for compound **23**.
